# 中文版莱斯特咳嗽问卷的改良及验证

**DOI:** 10.3779/j.issn.1009-3419.2017.07.05

**Published:** 2017-07-20

**Authors:** 嵘嘉 林, 国卫 车, 志华 徐, 明铭 王, 坤 周, 鹏飞 李

**Affiliations:** 610041 成都，四川大学华西医院胸外科 Department of Thoracic Surgery, West China Hospital, Sichuan University, Chengdu 610041, China

**Keywords:** 胸腔镜肺部手术, 莱斯特咳嗽问卷, 咳嗽, 肺部疾病, 信度, 效度, Video-assisted thoracic surgery, Leicester Cough Questionnaire in Mandarin-Chinese (LCQ-MC), Cough, Pulmonary disease, Reliability, Validity

## Abstract

**背景与目的:**

患者行肺部手术后常常出现咳嗽，目前尚缺乏专门评估术后咳嗽的工具。本研究对中文版莱斯特咳嗽问卷（Leicester Cough Questionnaire in Mandarin-Chinese, LCQ-MC）改良并进行验证，探讨其临床应用价值。

**方法:**

2015年9月-2016年12月四川大学华西医院胸外科单个医疗组共250例行胸腔镜肺部手术的患者参与调查，其中121例患者完成LCQ-MC，129例患者完成简化LCQ-MC，并进行信度和效度检验。

**结果:**

新问卷保留LCQ-MC的框架与评分方式，由生理、心理和社会3个维度，共12个条目构成。量表内容效度良好，内容效度指数达到0.83；与日间咳嗽症状积分对比标准效度高（*r*=-0.578, *P*＜0.001），与夜间咳嗽症状积分和健康调查简表总分（Chinese version of the Medical Outcome Study 36-item Short-Form Healthy Survey, SF-36）对比标准效度中等（*r*=-0.358, *P*=0.004; *r*=0.346, *P*=0.030），与医院焦虑与抑郁评分总分（Hospital Anxiety and Depression Scale, HADS）对比标准效度较弱（*r*=-0.241, *P*=0.046）；内部一致性良好，克朗巴赫α系数在0.71-0.84之间；1周后重测信度良好（*n*=30, *r*=0.81-0.95）。

**结论:**

简化版中文版莱斯特咳嗽问卷有良好的信度和效度，可应用于临床。

## Abstract (en)

**Background and objective:**

Patients often have cough after lung surgery, and there is a lack of tools to specifically assess postoperative coughs. LCQ-MC (Leicester Cough Questionnaire in Mandarin-Chinese) was revised and validated to explore its value on clinical application.

**Methods:**

A total of 250 patients undergone the lung operation of single medical team, from September 2015 to December 2016 in the Department ofThoracic Surgery, West China Hospital, Sichuan University, were investigated. Among them, 121 patients completed LCQ-MC and 129 patients completed simplified LCQ-MC, we verified the reliability and validity.

**Results:**

The new questionnaire was not changed in terms of content layout and the scoring method of LCQ-MC, consisting of 12 items and three domains (physical, psychological and social). There was good content validity (S-CVI/UA=0.83). Concurrent validity was high when the simplified LCQ-MC was compared with daytime cough symptom score (*r*=-0.578, *P* < 0.001). There was a moderate relationship with response to night-time cough symptom score (*r*=-0.358, *P*=-0.004) and SF36 total score (*r*=0.346, *P*=0.030), and weak relationship with the Hospital Anxiety and Depression Scale total score (*r*=-0.241, *P*=0.046). Cronbach's alpha coefficients of simplified LCQ-CM total and three domains varied between 0.79 and 0.89. One week apart test-retest reliability (*n*=30) was high (*r*=0.88-0.96).

**Conclusion:**

Simplified LCQ-MC has good reliability and validity that can be used for clinical applications.

术后症状恢复是评价快速康复的主要指标，而咳嗽是胸外科手术后常见的症状之一^[1, 2]^。研究^[3]^表明咳嗽是机体的防御反射，有利于清除呼吸道分泌物和有害因子，然而频繁剧烈的咳嗽对患者的机体造成损害，干扰日常生活甚至会加重心理负担。目前胸外科关于咳嗽评估工具应用的报道极少，尚缺乏专门评估咳嗽的工具^[4]^。本研究通过引入中文版莱斯特咳嗽问卷来评估肺部疾病患者术后咳嗽情况，并通过临床的应用对表格进行了改良并通过统计学验证，最终得到一个更加简介有效且适用于胸外科的评估工具^[5]^。

## 1 资料与方法

### 1.1 临床资料

被评估患者来自2015年9月-2016年12月四川大学华西医院胸外科单个医疗组，纳入标准：①年龄18岁-79岁；②术前签署知情同意书；③胸腔镜下肺叶、肺段或肺楔形肺部切除手术；④术后病理诊断明确。排除标准：①手术过程中转开胸、出血量大于1, 000 mL或二次手术；②全肺切除患者；③不同意参加调查或中途退出调查者。入选患者250例，男性111例，平均年龄55.77岁；女性139例，平均年龄54.93岁。其中行肺叶切除126例，肺段切除69例，楔形切除55例；术后诊断为肺癌167例，非肺癌83例。术后出现咳嗽121例，占48.4%。专家选取标准：①从事胸外科工作10年以上；②担任或曾担任医疗组长；③本科以上学历。共入选6位专家，参与量表的评议打分。

### 1.2 方法

#### 1.2.1 改良LCQ-MC的构建

本研究共通过2个阶段构建改良LCQ-MC，阶段1：2015年9月-2016年4月共121例患者完成LCQ-MC，通过数据分析与咨询专家意见，进行以下修改：①合并意思相近条目；②删除容易造成混淆条目；③使条目语言更通俗易懂，最终形成14个条目3个维度的LCQ-MC1（Leicester Cough Questionnaire in Mandarin-Chinese version 1），具体详见附件1。阶段2：将LCQ-MC1以问卷的形式亲自送达给专家，请专家就每一个条目与胸外科肺部疾病患者术后咳嗽情况的关联性予以评分，评分标准为“1分=不相关，2分=弱相关，3分=较强相关，4分=非常相关”，并提出修改建议。共送达6份问卷，回收6份。根据专家打分情况及建议修改，最终形成12个条目3个维度的LCQ-MC2（Leicester Cough Questionnaire in Mandarin-Chinese version 2），具体详见附件2。2016年6月-2016年12月共129例患者完成LCQ-MC2。阶段3：患者出院1周后重复调查一次LCQ-MC2，要求1周内仍有咳嗽症状且未使用相关控制咳嗽的药物，完成重复调查30份并计算重测信度。

#### 1.2.2 资料收集

参与调查的患者至少在2位经过专业培训的医护人员指导下完成问卷，填写时间一般为出院当天。术后需要使用药物控制咳嗽的患者，则在使用药物前完成问卷。

#### 1.2.3 对照量表的选用

本研究共选取3种量表做标准关联效度分析，分别是简化咳嗽症状积分、中文版医院焦虑与抑郁评分和中文版健康调查简表^[6-9]^。

### 1.3 统计学方法

本研究采用Excel 2013软件进行数据管理和SPSS 19.0软件进行统计学分析。采用克朗巴赫α系数和重测信度进行量表的信度分析，采用内容效度和标准关联效度进行量表的效度分析。

## 2 结果

### 2.1 效度分析

#### 2.1.1 内容效度

根据6位专家评议后结果，计算内容效度指数，在删除两个内容效度指数（item-level content validity index, I-CVI）小于0.83的条目后，形成LCQ-MC2。LCQ-MC2所有条目内容效度均大于0.83，且量表的内容效度（acale-level content validity index universal agreement, S-CVI/UA）由0.714提高至0.83，大于0.80，提示量表内容效度较好^[10]^。

#### 2.1.2 标准关联效度

一般认为*Pearson*相关系数*r*值绝对值大于0.5为强相关，0.3-0.5为中等相关，0.1-0.3为弱相关。LCQ-MC2总分与日间咳嗽症状积分的相关系数*r*值为-0.578（*P*＜0.001），呈强相关；与夜间咳嗽症状积分和SF-36总分中等相关（*r*=-0.358, *P*=0.004; *r*=0.346, *P*=0.030），与HADS总分弱相关（*r*=-0.241, *P*=0.046），均有统计学意义。说明LCQ-MC2标准关联效度较好，具体详见表 1。

**1:** LCQ-MC2的标准关联效度 Criterion-related validity of LCQ-MC2

	LCQ-MC2			Total
	Physical	Psychological	Social	
Daytime cough symptom score	-0.597	-0.508	-0.390	-0.578
Nighttime cough symptom score	-0.372	-0.284	-0.216	-0.358
HADS total score	-0.126	-0.208	-0.257	-0.241
SF-36 total score	0.123	0.560	0.321	0.346
LCQ-MC2: Leister Cough Questionnaire in Mandarin-Chinese version 2; HADS: Chinese version of the Hospital Anxiety and Depression Scale; SF-36: Chinese version of the Medical Outcome Study 36-item Short-Form Healthy Survey.				

### 2.2 信度分析

#### 2.2.1 内部一致性

LCQ-MC2的总体和三个维度的克朗巴赫α系数均大于0.7，与创建莱斯特咳嗽量表（Leister Cough Questionnaire, LCQ）的Birring等在慢性咳嗽患者、翻译LCQ-MC的Gao等在支气管炎患者中计算的克朗巴赫α系数相近，体现了LCQ-MC2的稳定性与良好的信度^[5, 11-13]^，具体详见表 2。

**2:** LCQ-MC2克朗巴赫*α*系数 Cronbach's alpha coefficient of LCQ-MC2

	Birring *et al*.^*^	Gao *et al*. ^+^	LCQ-MC2
Physical	0.79	0.83	0.71
Psychological	0.89	0.88	0.79
Social	0.85	0.82	0.71
Total	0.92	0.93	0.84
^*^Patients with chronic cough; ^+^Patients with non-cystic fibrosis bronchiectasis.			

#### 2.2.2 重测信度

通过30例患者出院后一周完成LCQ-MC2重复调查，各维度和总分的重复信度*r*均大于0.8，具体详见表 3，且与创建Birring等和Gao等计算的重测信度相近，说明LCQ-MC2的重复信度良好，可重复性高^[5, 13-15]^。

**3:** 1周后LCQ-MC2重测信度 Test-retest reliability of LCQ-MC2 (one week apart)

	Birring *et al*.^*^	Gao *et al*. ^+^	LCQ-MC2
Physical	0.93	0.84	0.95
Psychological	0.90	0.82	0.81
Social	0.88	0.89	0.85
Total	0.96	0.89	0.88
^*^Patients with chronic cough; ^+^Patients with non-cystic fibrosis bronchiectasis.						

## 3 讨论

外科手术后，患者常常出现不同程度的咳嗽，这可能与手术创伤、麻醉或者气管插管有关^[16, 17]^。胸外科手术由于其手术与疾病的特殊性，术后出现的咳嗽几率较高且持续时间较长，有些甚至会演变成顽固性咳嗽，严重影响患者的术后康复与生活质量^[18]^。然而，胸外科缺乏能客观有效评估咳嗽的工具，不利于术后咳嗽的临床研究以及加速患者术后康复^[4, 19, 20]^。因此，研制一种适用于胸外科术后咳嗽的评估工具有重要临床意义。

本研究所采用LCQ-MC，最早由Birring等于2003年创建，2009年由Wei等首次翻译为中文，2014年由Gao等正式翻译成普通话版使其更加符合中国人使用习惯；该量表由19个条目，社会、心理和社会3个维度组成，7个等级正项计分^[5, 13, 21]^。但是，国内关于LCQ-MC应用于胸外科的报道较少，本研究首次将LCQ-MC引入用于胸外科评估肺疾病患者术后咳嗽情况^[4]^。在本研究开始阶段，直接引用LCQ-MC用于调查肺部疾病患者术后咳嗽情况，通过临床实践中的问题与专家的建议将LCQ-MC初步修减为14个条目。在随后的验证阶段中，逐步将量表缩减为12个条目，得到最终版本的问卷。新问卷保留了LCQ-MC的结构与评分方式，并对语言进行了进一步优化，比如将原条目中的“2周内”，统一修改为“手术后”，使其更加适合外科使用。

经过统计学验证后的新量表具有良好的信度与效度。效度分析主要通过内容效度与标准关联效度验证：内容效度指量表各条目是否测定其希望的内容，一般通过专家评议打分，新问卷的所有条目内容效度（I-CVI）均大于0.83，内容效度（S-CVI/UA）达到0.83，说明了其新量表的内容能较好地反映出肺部疾病患者术后的咳嗽情况^[10, 22, 23]^。标准关联效度选用了目前较为公认的三个评价工具加以比较，其中使用日间夜间症状咳嗽积分为中国2009年版咳嗽指南提供的简化版，但仅为专家共识，尚未经过严格验证；中文版HADS主要应用于综合医院患者中焦虑和抑郁的筛查，是目前临床上最常见的症状自评工具；中文版SF-36是全球应用最广的生命质量测试工具^[6-9, 24]^。新量表总分与日间咳嗽症状积分强相关、夜间咳嗽症状积分中等相关，与SF-36总分中等相关；与HADS弱相关，说明新量表能较好地体现患者在生理和社会维度方面的健康变化情况，但是对于心理方面的变化较差，可能是由于心理维度由原来7个条目缩减为3个条目导致。信度分析主要通过内部一致性与重测信度验证：采用克朗巴赫α系数，计算出的值越高说明内部一致性越好，一般认为大于0.7就能说明有较好的信度，新问卷的总分和各维度克朗巴赫α系数介于0.71-0.84^[11, 12]^。30例患者在出院后一周完成了重复调查，重测信度介于0.81-0.95，说明量表的重复性高^[14, 15]^。

本研究尚存在许多不足之处，不利简化中文版莱斯特咳嗽问卷在胸外科的推广。首先，简化中文版莱斯特咳嗽问卷调查对象为胸外科肺部疾病患者，未详细区分肺癌患者与非肺癌患者，在今后的研究中应将肺癌患者与非肺癌患者分开研究^[25]^。其次，由于样本量过少，未能计算最小临床差异值（minimal clinically important difference, MCID），亟需进一步的研究^[26, 27]^。最后，对于是否继续沿用LCQ-MC的评分方式和三个维度还有待进一步讨论。

综上所述，简化中文版莱斯特咳嗽问卷在胸外科的应用中取得了良好的效果，但是也存在一些不足之处，相信在未来的不断应用与改进中，新问卷能成为一种简洁、有效的咳嗽评估工具。
